# Bis{μ-2-[1-(2-Pyridylmethyl­imino)eth­yl]phenolato}bis­[azido­copper(II)]

**DOI:** 10.1107/S1600536809030475

**Published:** 2009-08-26

**Authors:** Jun Zhang, Xiao-Dan Chen, Huai-Hong Zhang, Bai-Wang Sun

**Affiliations:** a Ordered Matter Science Research Center, College of Chemistry and Chemical Engineering, Southeast University, Nanjing 210096, People’s Republic of China

## Abstract

The title compound, [Cu_2_(C_14_H_13_N_2_O)_2_(N_3_)_2_], was synthesized by the reaction of Cu(NO_3_)_2_·3H_2_O with the Schiff base 2-[1-(2-pyridylmethyl­imino)eth­yl]phenol (H*L*) in methanol–water solution, adding NaN_3_ as the bridging ligand. The asymmetric unit contains one half-mol­ecule, the other half being generated by the inversion center. Each Cu^II^ atom shows a slightly distorted trigonal-pyramidal geometry formed by two N atoms and one O atom from one Schiff base ligand, by another O atom of a second Schiff base ligand and by an azide N atom. The crystal structure is stabilized by intermolecular C—H⋯N hydrogen bonds.

## Related literature

For the potential applications in catalysis and enzymatic reactions, magnetism and mol­ecular architecture of transition metal compounds containing Schiff base ligands, see: Li & Zhang (2004 ▶); You & Zhu (2004 ▶). For the synthesis, see: Pointeau *et al.* (1986 ▶).
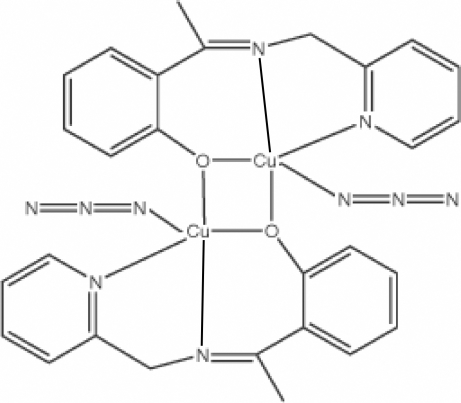


## Experimental

### 

#### Crystal data

[Cu_2_(C_14_H_13_N_2_O)_2_(N_3_)_2_]
*M* *_r_* = 661.67
Monoclinic, 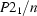

*a* = 10.1066 (12) Å
*b* = 8.0545 (10) Å
*c* = 16.7027 (18) Å
β = 96.251 (1)°
*V* = 1351.6 (3) Å^3^
*Z* = 2
Mo *K*α radiation
μ = 1.62 mm^−1^
*T* = 298 K
0.20 × 0.12 × 0.09 mm

#### Data collection

Rigaku SCXmini diffractometer
Absorption correction: multi-scan (*CrystalClear*; Rigaku, 2005 ▶) *T*_min_ = 0.737, *T*_max_ = 0.868
6641 measured reflections
2379 independent reflections
1720 reflections with *I* > 2σ(*I*)
*R*_int_ = 0.042

#### Refinement

*R*[*F* ^2^ > 2σ(*F* ^2^)] = 0.035
*wR*(*F* ^2^) = 0.062
*S* = 1.02
2379 reflections
190 parameters
H-atom parameters constrained
Δρ_max_ = 0.35 e Å^−3^
Δρ_min_ = −0.33 e Å^−3^

### 

Data collection: *CrystalClear* (Rigaku, 2005 ▶); cell refinement: *CrystalClear*; data reduction: *CrystalClear*; program(s) used to solve structure: *SHELXS97* (Sheldrick, 2008 ▶); program(s) used to refine structure: *SHELXL97* (Sheldrick, 2008 ▶); molecular graphics: *SHELXTL* (Sheldrick, 2008 ▶); software used to prepare material for publication: *SHELXL97*.

## Supplementary Material

Crystal structure: contains datablocks I, global. DOI: 10.1107/S1600536809030475/at2842sup1.cif

Structure factors: contains datablocks I. DOI: 10.1107/S1600536809030475/at2842Isup2.hkl

Additional supplementary materials:  crystallographic information; 3D view; checkCIF report

## Figures and Tables

**Table 1:** Hydrogen-bond geometry (Å, °)

*D*—H⋯*A*	*D*—H	H⋯*A*	*D*⋯*A*	*D*—H⋯*A*
C9—H9*B*⋯N5^i^	0.97	2.55	3.399 (5)	147
C14—H14⋯N3	0.93	2.55	3.052 (4)	114

Symmetry code: (i) 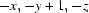.

**Fig. 1.**
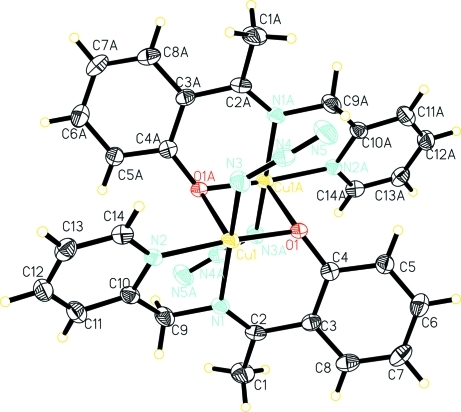
The molecular structure of the title compound with the atom-numbering scheme. Displacement ellipsoids are drawn at the 30% probability level.

## supplementary crystallographic information

### Comment

Transition metal compounds containing Schiff base ligands have been of great interest since many years. These compounds play an important role in the development of coordination chemistry related to their potential applications in catalysis and enzymatic reactions, magnetism and molecular architecture (You & Zhu, 2004; Li & Zhang, 2004). We have focused on the synthesis of Schiff base complexes which is formed by 2-(pyridin-2-ylethyliminomethyl)phenol (HL1) and some metal salts. To enrich our studies on schiff bases, we used HL (Pointeau, *et al.*, 1986) instead of HL1 and gained the title compound. So, we reported this dinuclear copper(II) complex here.

The structure analyses show that complex crystallizes in monoclinic space group P21/n. The asymmetric unit contains only half of the unique molecule, and the other half is related by the inversion center (Fig.1). The molecule of the title compound is composed of two Cu^II^ atoms, two schiff base ligand 2-[1-(pyridin-2-ylmethylimino)-ethyl]-phenol and two azidos. Each Cu^II^ atom shows a slightly distorted trigonal-bipyramidal geometry formed by two N atoms and one O atom from one schiff base ligand (You & Zhu, 2004), the another O atom of the second schiff base, together with another N atom from azido.

In the structure, there are intra and intermolecular C—H···N hydrogen bond interactions (Table 2).

### Experimental

The title compound was synthesized by Cu(NO_3_)_2_.3H_2_O, schiff base ligand 2-[1-(pyridin-2'-ylmethylimino)-ethyl]-phenol and sodium azide. All chemicals used (reagent grade) were commercially available. 2'-hydroxyacetophenone(0.136 g, 1 mmol) was dissolved in ethanol (5 mL) and ethanol solution (5 ml) containing 2-aminoethylpyri dine (0.108 g, 1 mmol) was added slowly with stirring. The resulting yellow solution was continuously stirred for about 30 min. at room temperature, and then Cu(NO_3_)_2_.3H_2_O (0.241 g, 1 mmol) and sodium azide (0.13 g, 2 mmol) in aqueous solution (5 ml) was added with stirring homogeneously. Brown crystals suitable for X-ray analysis were obtained by slow evaporation at room temperature over several days.

### Refinement

H atoms bound to carbon were placed in geometrical positions and refined using a riding model, with C—H = 0.93-0.97Å and *U*_iso_(H) =1.2 or 1.5*U*_eq_(C).

### Crystal data

**Table:**

[Cu_2_(C_14_H_13_N_2_O)_2_(N_3_)_2_]	*F*(000) = 676
*M**_r_* = 661.67	*D*_x_ = 1.626 Mg m^−^^3^
Monoclinic, *P*2_1_/*n*	Mo *K*α radiation, λ = 0.71073 Å
Hall symbol: -P 2yn	Cell parameters from 13380 reflections
*a* = 10.1066 (12) Å	θ = 3.0–27.6°
*b* = 8.0545 (10) Å	µ = 1.62 mm^−^^1^
*c* = 16.7027 (18) Å	*T* = 298 K
β = 96.251 (1)°	Prism, dark green
*V* = 1351.6 (3) Å^3^	0.20 × 0.12 × 0.09 mm
*Z* = 2	

### Data collection

**Table:**

Rigaku SCXmini diffractometer	2379 independent reflections
Radiation source: fine-focus sealed tube	1720 reflections with *I* > 2σ(*I*)
graphite	*R*_int_ = 0.042
Detector resolution: 8.192 pixels mm^-1^	θ_max_ = 25.0°, θ_min_ = 2.3°
Thin–slice ω scans	*h* = −12→7
Absorption correction: multi-scan (*CrystalClear*; Rigaku, 2005)	*k* = −9→9
*T*_min_ = 0.737, *T*_max_ = 0.868	*l* = −19→19
6641 measured reflections	

### Refinement

**Table:**

Refinement on *F*^2^	Primary atom site location: structure-invariant direct methods
Least-squares matrix: full	Secondary atom site location: difference Fourier map
*R*[*F*^2^ > 2σ(*F*^2^)] = 0.035	Hydrogen site location: inferred from neighbouring sites
*wR*(*F*^2^) = 0.062	H-atom parameters constrained
*S* = 1.02	*w* = 1/[σ^2^(*F*_o_^2^) + (0.0192*P*)^2^] where *P* = (*F*_o_^2^ + 2*F*_c_^2^)/3
2379 reflections	(Δ/σ)_max_ < 0.001
190 parameters	Δρ_max_ = 0.35 e Å^−^^3^
0 restraints	Δρ_min_ = −0.33 e Å^−^^3^

### Special details

**Table:**

Geometry. All e.s.d.'s (except the e.s.d. in the dihedral angle between two l.s. planes) are estimated using the full covariance matrix. The cell e.s.d.'s are taken into account individually in the estimation of e.s.d.'s in distances, angles and torsion angles; correlations between e.s.d.'s in cell parameters are only used when they are defined by crystal symmetry. An approximate (isotropic) treatment of cell e.s.d.'s is used for estimating e.s.d.'s involving l.s. planes.
Refinement. Refinement of *F*^2^ against ALL reflections. The weighted *R*-factor *wR* and goodness of fit *S* are based on *F*^2^, conventional *R*-factors *R* are based on *F*, with *F* set to zero for negative *F*^2^. The threshold expression of *F*^2^ > σ(*F*^2^) is used only for calculating *R*-factors(gt) *etc*. and is not relevant to the choice of reflections for refinement. *R*-factors based on *F*^2^ are statistically about twice as large as those based on *F*, and *R*- factors based on ALL data will be even larger.

### Fractional atomic coordinates and isotropic or equivalent isotropic displacement parameters (Å^2^)

**Table:**

	*x*	*y*	*z*	*U*_iso_*/*U*_eq_	
Cu1	0.00744 (4)	0.69734 (4)	0.00638 (2)	0.03343 (13)	
N1	−0.0002 (2)	0.6887 (3)	0.12413 (13)	0.0349 (6)	
N2	−0.1427 (2)	0.8584 (3)	0.01237 (14)	0.0324 (6)	
N3	0.0263 (3)	0.7564 (3)	−0.10588 (15)	0.0448 (7)	
N4	0.1145 (3)	0.7080 (3)	−0.14292 (14)	0.0391 (7)	
N5	0.1975 (3)	0.6669 (4)	−0.18111 (16)	0.0579 (9)	
O1	0.14375 (17)	0.5289 (2)	0.01510 (10)	0.0333 (5)	
C1	0.0637 (3)	0.6042 (5)	0.26461 (17)	0.0594 (10)	
H1A	0.1024	0.6932	0.2975	0.089*	
H1B	0.1029	0.5008	0.2834	0.089*	
H1C	−0.0305	0.6009	0.2678	0.089*	
C2	0.0898 (3)	0.6322 (4)	0.17764 (17)	0.0374 (8)	
C3	0.2216 (3)	0.5861 (4)	0.15511 (17)	0.0359 (8)	
C4	0.2396 (3)	0.5298 (4)	0.07618 (17)	0.0347 (8)	
C5	0.3666 (3)	0.4732 (4)	0.06349 (19)	0.0417 (8)	
H5	0.3788	0.4258	0.0141	0.050*	
C6	0.4741 (3)	0.4850 (4)	0.1214 (2)	0.0530 (9)	
H6	0.5573	0.4477	0.1104	0.064*	
C7	0.4584 (4)	0.5521 (4)	0.1957 (2)	0.0583 (11)	
H7	0.5314	0.5661	0.2340	0.070*	
C8	0.3338 (4)	0.5977 (4)	0.21236 (19)	0.0482 (9)	
H8	0.3230	0.6379	0.2634	0.058*	
C9	−0.1311 (3)	0.7385 (4)	0.14550 (18)	0.0443 (9)	
H9A	−0.1217	0.7914	0.1980	0.053*	
H9B	−0.1871	0.6413	0.1483	0.053*	
C10	−0.1947 (3)	0.8571 (4)	0.08326 (18)	0.0366 (8)	
C11	−0.2990 (3)	0.9584 (4)	0.0981 (2)	0.0496 (10)	
H11	−0.3332	0.9548	0.1475	0.060*	
C12	−0.3517 (3)	1.0650 (4)	0.0388 (2)	0.0528 (10)	
H12	−0.4217	1.1350	0.0478	0.063*	
C13	−0.3000 (3)	1.0671 (4)	−0.0340 (2)	0.0473 (9)	
H13	−0.3352	1.1373	−0.0752	0.057*	
C14	−0.1951 (3)	0.9631 (4)	−0.04483 (19)	0.0421 (8)	
H14	−0.1593	0.9658	−0.0938	0.050*	

### Atomic displacement parameters (Å^2^)

**Table:**

	*U*^11^	*U*^22^	*U*^33^	*U*^12^	*U*^13^	*U*^23^
Cu1	0.0379 (2)	0.0380 (2)	0.0248 (2)	0.0016 (2)	0.00541 (15)	−0.00083 (19)
N1	0.0380 (16)	0.0401 (16)	0.0275 (14)	−0.0004 (13)	0.0078 (11)	−0.0019 (13)
N2	0.0352 (16)	0.0305 (15)	0.0317 (15)	−0.0032 (11)	0.0049 (12)	−0.0038 (11)
N3	0.0442 (18)	0.061 (2)	0.0314 (15)	0.0104 (14)	0.0125 (13)	0.0084 (13)
N4	0.0471 (19)	0.0414 (17)	0.0282 (15)	−0.0024 (15)	0.0017 (13)	0.0035 (13)
N5	0.063 (2)	0.066 (2)	0.0480 (18)	0.0109 (17)	0.0234 (16)	−0.0012 (16)
O1	0.0343 (12)	0.0404 (14)	0.0245 (11)	0.0027 (10)	0.0009 (9)	−0.0022 (9)
C1	0.075 (3)	0.076 (3)	0.029 (2)	0.009 (2)	0.0094 (17)	0.0074 (18)
C2	0.053 (2)	0.0332 (19)	0.0261 (18)	−0.0024 (16)	0.0044 (16)	−0.0019 (14)
C3	0.042 (2)	0.0366 (19)	0.0282 (18)	−0.0008 (16)	−0.0008 (15)	0.0049 (14)
C4	0.0376 (19)	0.032 (2)	0.0349 (19)	−0.0034 (14)	0.0051 (15)	0.0039 (14)
C5	0.039 (2)	0.047 (2)	0.0389 (19)	0.0029 (17)	0.0041 (15)	0.0007 (16)
C6	0.039 (2)	0.060 (2)	0.058 (2)	0.0060 (19)	−0.0008 (17)	0.006 (2)
C7	0.047 (2)	0.069 (3)	0.053 (3)	−0.0019 (19)	−0.0188 (18)	0.004 (2)
C8	0.061 (3)	0.049 (2)	0.033 (2)	−0.0034 (19)	−0.0043 (17)	0.0018 (16)
C9	0.049 (2)	0.053 (2)	0.0342 (19)	0.0045 (17)	0.0166 (16)	0.0016 (16)
C10	0.038 (2)	0.035 (2)	0.037 (2)	−0.0054 (15)	0.0083 (15)	−0.0065 (15)
C11	0.048 (2)	0.051 (3)	0.051 (2)	0.0052 (18)	0.0162 (18)	−0.0071 (18)
C12	0.042 (2)	0.052 (2)	0.065 (3)	0.0054 (18)	0.0059 (19)	−0.016 (2)
C13	0.044 (2)	0.039 (2)	0.056 (2)	−0.0018 (17)	−0.0072 (17)	0.0015 (17)
C14	0.046 (2)	0.042 (2)	0.038 (2)	−0.0022 (16)	0.0022 (15)	−0.0026 (16)

### Geometric parameters (Å, °)

**Table:**

Cu1—O1	1.9278 (18)	C4—C5	1.399 (4)
Cu1—N3	1.964 (2)	C5—C6	1.377 (4)
Cu1—N1	1.978 (2)	C5—H5	0.9300
Cu1—N2	2.007 (2)	C6—C7	1.379 (4)
Cu1—O1^i^	2.3799 (19)	C6—H6	0.9300
N1—C2	1.287 (4)	C7—C8	1.369 (4)
N1—C9	1.463 (3)	C7—H7	0.9300
N2—C14	1.340 (4)	C8—H8	0.9300
N2—C10	1.347 (3)	C9—C10	1.503 (4)
N3—N4	1.204 (3)	C9—H9A	0.9700
N4—N5	1.156 (3)	C9—H9B	0.9700
O1—C4	1.328 (3)	C10—C11	1.377 (4)
O1—Cu1^i^	2.3799 (19)	C11—C12	1.373 (4)
C1—C2	1.521 (4)	C11—H11	0.9300
C1—H1A	0.9600	C12—C13	1.375 (4)
C1—H1B	0.9600	C12—H12	0.9300
C1—H1C	0.9600	C13—C14	1.378 (4)
C2—C3	1.471 (4)	C13—H13	0.9300
C3—C8	1.404 (4)	C14—H14	0.9300
C3—C4	1.424 (4)		
			
O1—Cu1—N3	95.73 (9)	C5—C4—C3	117.1 (3)
O1—Cu1—N1	90.30 (9)	C6—C5—C4	122.5 (3)
N3—Cu1—N1	167.49 (11)	C6—C5—H5	118.7
O1—Cu1—N2	171.51 (8)	C4—C5—H5	118.7
N3—Cu1—N2	92.50 (10)	C5—C6—C7	120.0 (3)
N1—Cu1—N2	82.03 (10)	C5—C6—H6	120.0
O1—Cu1—O1^i^	85.08 (7)	C7—C6—H6	120.0
N3—Cu1—O1^i^	99.74 (9)	C8—C7—C6	119.1 (3)
N1—Cu1—O1^i^	91.66 (8)	C8—C7—H7	120.4
N2—Cu1—O1^i^	91.47 (8)	C6—C7—H7	120.4
C2—N1—C9	121.1 (2)	C7—C8—C3	122.4 (3)
C2—N1—Cu1	127.0 (2)	C7—C8—H8	118.8
C9—N1—Cu1	111.55 (18)	C3—C8—H8	118.8
C14—N2—C10	118.1 (3)	N1—C9—C10	109.6 (2)
C14—N2—Cu1	127.9 (2)	N1—C9—H9A	109.8
C10—N2—Cu1	114.1 (2)	C10—C9—H9A	109.8
N4—N3—Cu1	124.5 (2)	N1—C9—H9B	109.8
N5—N4—N3	176.9 (3)	C10—C9—H9B	109.8
C4—O1—Cu1	120.63 (17)	H9A—C9—H9B	108.2
C4—O1—Cu1^i^	121.52 (17)	N2—C10—C11	122.2 (3)
Cu1—O1—Cu1^i^	94.91 (7)	N2—C10—C9	115.8 (3)
C2—C1—H1A	109.5	C11—C10—C9	122.0 (3)
C2—C1—H1B	109.5	C12—C11—C10	119.1 (3)
H1A—C1—H1B	109.5	C12—C11—H11	120.5
C2—C1—H1C	109.5	C10—C11—H11	120.5
H1A—C1—H1C	109.5	C11—C12—C13	119.4 (3)
H1B—C1—H1C	109.5	C11—C12—H12	120.3
N1—C2—C3	120.2 (3)	C13—C12—H12	120.3
N1—C2—C1	122.2 (3)	C12—C13—C14	118.7 (3)
C3—C2—C1	117.6 (3)	C12—C13—H13	120.7
C8—C3—C4	118.5 (3)	C14—C13—H13	120.7
C8—C3—C2	119.7 (3)	N2—C14—C13	122.6 (3)
C4—C3—C2	121.9 (3)	N2—C14—H14	118.7
O1—C4—C5	119.1 (3)	C13—C14—H14	118.7
O1—C4—C3	123.8 (3)		
			
O1—Cu1—N1—C2	20.2 (3)	C1—C2—C3—C4	149.0 (3)
N3—Cu1—N1—C2	−98.8 (6)	Cu1—O1—C4—C5	−146.9 (2)
N2—Cu1—N1—C2	−163.5 (3)	Cu1^i^—O1—C4—C5	94.4 (3)
O1^i^—Cu1—N1—C2	105.2 (3)	Cu1—O1—C4—C3	32.9 (4)
O1—Cu1—N1—C9	−153.2 (2)	Cu1^i^—O1—C4—C3	−85.7 (3)
N3—Cu1—N1—C9	87.8 (5)	C8—C3—C4—O1	−173.3 (3)
N2—Cu1—N1—C9	23.2 (2)	C2—C3—C4—O1	6.6 (5)
O1^i^—Cu1—N1—C9	−68.1 (2)	C8—C3—C4—C5	6.5 (4)
N3—Cu1—N2—C14	−2.0 (3)	C2—C3—C4—C5	−173.6 (3)
N1—Cu1—N2—C14	166.7 (3)	O1—C4—C5—C6	173.6 (3)
O1^i^—Cu1—N2—C14	−101.8 (2)	C3—C4—C5—C6	−6.2 (5)
N3—Cu1—N2—C10	177.8 (2)	C4—C5—C6—C7	1.1 (5)
N1—Cu1—N2—C10	−13.5 (2)	C5—C6—C7—C8	3.6 (5)
O1^i^—Cu1—N2—C10	78.0 (2)	C6—C7—C8—C3	−3.1 (5)
O1—Cu1—N3—N4	−9.5 (3)	C4—C3—C8—C7	−2.1 (5)
N1—Cu1—N3—N4	109.0 (5)	C2—C3—C8—C7	178.0 (3)
N2—Cu1—N3—N4	172.6 (3)	C2—N1—C9—C10	158.1 (3)
O1^i^—Cu1—N3—N4	−95.5 (3)	Cu1—N1—C9—C10	−28.1 (3)
N3—Cu1—O1—C4	129.3 (2)	C14—N2—C10—C11	0.3 (4)
N1—Cu1—O1—C4	−39.7 (2)	Cu1—N2—C10—C11	−179.6 (2)
O1^i^—Cu1—O1—C4	−131.4 (2)	C14—N2—C10—C9	−179.4 (3)
N3—Cu1—O1—Cu1^i^	−99.33 (9)	Cu1—N2—C10—C9	0.8 (3)
N1—Cu1—O1—Cu1^i^	91.64 (8)	N1—C9—C10—N2	17.9 (4)
O1^i^—Cu1—O1—Cu1^i^	0.0	N1—C9—C10—C11	−161.8 (3)
C9—N1—C2—C3	−178.8 (3)	N2—C10—C11—C12	−0.2 (5)
Cu1—N1—C2—C3	8.5 (4)	C9—C10—C11—C12	179.4 (3)
C9—N1—C2—C1	3.6 (5)	C10—C11—C12—C13	0.5 (5)
Cu1—N1—C2—C1	−169.1 (2)	C11—C12—C13—C14	−0.9 (5)
N1—C2—C3—C8	151.1 (3)	C10—N2—C14—C13	−0.7 (4)
C1—C2—C3—C8	−31.2 (4)	Cu1—N2—C14—C13	179.1 (2)
N1—C2—C3—C4	−28.7 (4)	C12—C13—C14—N2	1.0 (5)

Symmetry codes: (i) −*x*, −*y*+1, −*z*.

### Hydrogen-bond geometry (Å, °)

**Table:**

*D*—H···*A*	*D*—H	H···*A*	*D*···*A*	*D*—H···*A*
C9—H9B···N5^i^	0.97	2.55	3.399 (5)	147
C14—H14···N3	0.93	2.55	3.052 (4)	114

Symmetry codes: (i) −*x*, −*y*+1, −*z*.

## References

[bb1] Li, Z.-X. & Zhang, X.-L. (2004). *Acta Cryst.* E**60**, m1017–m1019.

[bb2] Pointeau, P., Patin, H., Mousser, A. & le Marouile, J.-Y. (1986). *J. Organomet. Chem.* **312**, 263–276.

[bb3] Rigaku. (2005). *CrystalClear* Rigaku Corporation, Tokyo, Japan.

[bb4] Sheldrick, G. M. (2008). *Acta Cryst.* A**64**, 112–122. 10.1107/S0108767307043930 18156677

[bb5] You, Z.-L. & Zhu, H.-L. (2004). *Z. Anorg. Allg. Chem.* **630**, 2754–2760.

